# Aktuelle Herausforderungen im Erwachsenenschutzrecht und damit verbundene Anforderungen an die Soziale Arbeit

**DOI:** 10.1007/s12592-022-00412-w

**Published:** 2022-05-09

**Authors:** Anika Gomille

**Affiliations:** Fakultät III, Wirtschaftsrecht, Universität Siegen, Siegen, Deutschland

**Keywords:** Rechtswirklichkeit, Sozialpädagogik, Betreuungsrecht, Corona-Pandemie, Rechtssoziologie, Law in action, Social pedagogy, Guardianship law, Corona pandemic, Sociology of law

## Abstract

Mit der zweiten Moderne sind gesellschaftliche Veränderungen einhergegangen, die neue Anforderungen an die Soziale Arbeit stellen. Dies betrifft auch den Bereich des Erwachsenenschutzrechts, in dem der Gesetzgeber in den letzten Jahren verschiedene rechtliche Regelungen für bestimmte Zielgruppen geschaffen hat. Die damit verbundenen Herausforderungen für die Soziale Arbeit, die sich durch die aktuelle Corona-Pandemie noch verschärfen, werden im folgenden Artikel dargestellt und die Anforderungen anhand der einzelnen Bereiche differenziert untersucht. Mit einer heuristischen Betrachtung der gesellschaftlichen Entwicklungen und ihrer Folgeeffekte lassen sich zukünftige Anforderungen prognostizieren.

## Abstract (en)

The second modern age has been accompanied by societal changes that place new demands on social work. These changes also affect the field of adult protection law, in which the legislature has created various legal regulations for specific target groups in recent years. The following article presents the associated challenges for social work, which are exacerbated by the current coronavirus pandemic, and examines the requirements in a differentiated approach based on the individual areas. A heuristic view of social developments and their subsequent effects can be applied to forecast future requirements.

Die eine Form der genuin klassischen Sozialen Arbeit existiert nicht. Das Spektrum an Tätigkeitsfeldern, die darunter gefasst werden können, ist breit, die an die Fachkräfte gestellten Anforderungen sind vielfältig. Die Annahme liegt nahe, dass sich diese Situation auch in Zukunft nicht ändern, sondern eher verschärfen wird. Inwieweit mit COVID-19 und der aktuellen pandemischen Situation zusätzliche, auf Dauer angelegte Herausforderungen für den Bereich der Sozialen Arbeit verbunden sind, lässt sich nicht genau vorhersagen. Durch eine professionssoziologische Perspektive lassen sich jedoch die Entwicklungen aktueller Herausforderungen zumindest retrospektiv beleuchten und auf dieser Grundlage Erkenntnisse über mögliche Entwicklungen und Anforderungen für die sozialarbeiterische Praxis beschreiben.

Der Artikel befasst sich zu diesem Zweck exemplarisch mit den neuen Herausforderungen im speziellen Feld des Erwachsenenschutzrechts. Dafür liegt ihm ein weiter Begriff des Erwachsenenschutzrechts zugrunde, der nicht nur die gleichberechtigte Teilhabe von Menschen mit einer Behinderung umfasst, sondern auch für jegliche erwachsene, schutz- und hilfebedürftige Person offen ist. Einem rechtssoziologischen Verständnis folgend, wird der Fokus nicht allein auf die Summe gesetzlicher Regelungen in diesem Bereich gesetzt, sondern er richtet sich insbesondere auf die Wechselwirkungen zwischen rechtlichen Anforderungen auf der einen und praktischen Anforderungen, wie sie sich an die Soziale Arbeit richten, auf der anderen Seite. Gerade in diesem Zusammenhang, so die leitende Annahme, zeigt sich bei einer heuristischen Betrachtung eine Vielzahl von mit der zweiten Moderne verbundenen Veränderungen, die den rechtlichen Bezugsrahmen sozialarbeiterischer Tätigkeit schon heute prägen, aber auch zukünftig weiter verändern werden. Der vorliegende Beitrag fokussiert die Anforderungen, die sich für die Soziale Arbeit im Feld des Erwachsenschutzes durch eine gestiegene Bedeutung der Selbstbestimmung als Folge der Individualisierung ergeben haben. Ausgehend von einer steigenden Bedeutung des Erwachsenschutzrechts wird anhand ausgewählter Themenkomplexe zunächst die Erweiterung von Handlungsoptionen und Lebensformen der Individuen der zweiten Moderne beschrieben. Ein Blick auf unterschiedliche Lebensbereiche veranschaulicht konkrete Veränderungen und zeigt, dass der Trend zur Individualisierung auch auf rechtlicher Ebene erkennbar ist. Die praktischen Anforderungen an erfolgreiche Soziale Arbeit im Erwachsenenschutzrecht lassen sich nicht ohne diese rechtliche Rahmung verstehen. Eine Unsicherheit kann sich hier vor allem durch die stetig fortschreitende Rechtsentwicklung ergeben, die juristische Kenntnisse unabdingbar macht. Gleichzeitig lässt sich die fortschreitende Rechtsentwicklung aber nicht losgelöst von sozialwissenschaftlichem Wissen begreifen.

## Die (steigende) Bedeutung des Erwachsenenschutzrechts

Zu einer näheren Betrachtung des Erwachsenenschutzes sind verschiedene Anknüpfungspunkte vorhanden. So kann unter anderem auf den Adressatenkreis fokussiert werden. Nach dem Haager Erwachsenenschutzabkommen sind die Träger*innen der vorgesehenen Rechte all jene Erwachsene, die aufgrund einer Beeinträchtigung oder der Unzulänglichkeit ihrer persönlichen Fähigkeiten nicht allein in der Lage sind, ihre Interessen zu schützen und ihre Rechte wahrzunehmen (Art. 1 Abs. 1 ErwSÜ). Betroffene Rechtsgebiete mit entsprechend unterschiedlichen Perspektiven, so z. B. Verfassungsrecht, Völkerrecht oder aber Sozial- oder Betreuungsrecht, können ebenfalls den Ausgangspunkt einer analytischen Betrachtung darstellen. Ein weiterer Weg, sich der Frage nach der Bedeutung des Erwachsenenschutzrechts zu stellen, ergibt sich durch die Reflexion über den Gegenstand und hier insbesondere durch die Umsetzung des Rechts in der alltäglichen Praxis. Aus einer rechtssoziologischen Perspektive zeigt sich, dass Erwachsenenschutzrechte nicht mehr (aber auch nicht weniger) sind als Zweckprogramme des Rechts. Das bedeutet, dass sie als bloße Worthülsen *leerlaufen*, wenn sie nicht gefüllt werden. Im Gegensatz zu Konditionalprogrammen geht es beim Erwachsenenschutzrecht um die rein rechtliche Vorgabe von Ergebnissen, die zu erreichen sind. Es handelt sich bei den entsprechenden Normen also lediglich um die Vorgabe von Zielen, die anzustreben sind, wobei die konkrete Festlegung des Weges zur Zielerreichung den Praktikern im Feld überlassen ist (Luhmann, 1969, S. 129 ff.).

Eine Besonderheit der sozialarbeiterischen Tätigkeit lässt sich daher in der gestaltenden Aufgabe und Herausforderung sehen, diesen rechtlichen Zweckprogrammen als bloßen Worthülsen Leben einzuhauchen und sie stetig durch ihre Arbeit mit Inhalt zu füllen.

Eine gewisse Reglementierung ergibt sich durch die strukturelle Einbindung der Profession. Als praktizierende Instanz liegt die Soziale Arbeit dabei auf struktureller Ebene mittig, d. h. fachlich oberhalb der Polizei, aber unterhalb der Gerichte. Dabei ergaben sich seit jeher und ergeben sich zunehmend mehr Schnittstellen, die eine verstärkte Zusammenarbeit untereinander erfordern. Inhaltliche Einschränkungen ergeben sich auch dadurch, dass die rechtlichen Grundlagen im Einklang mit gesellschaftlichen Erfordernissen stehen sollten.^1^

Das lässt sich z. B. anhand der Entwicklung des Jugendstrafrechts gut veranschaulichen. So zeigt ein Blick in die Vergangenheit, dass das Jugendgerichtsgesetz ebenfalls mit Veränderungen konfrontiert wurde, die Auswirkungen auf das Feld der Sozialen Arbeit hatten. Die Überlegung, dass es zum Schutz von Kindern und Jugendlichen, zum Teil auch von Heranwachsenden, einer unabhängigen sozialen Instanz bedürfe, die das Defizit dieser Zielgruppen, das gerade gegenüber Erwachsenen vor Gericht besteht, ausgleicht, führte einst zur Schaffung der Jugendgerichtshilfe (vgl. Kruse 1937, S. 523 ff.). Ein Implementationsgedanke, der dazu führte, dass sich heute Zwischenstellungen für Personen ergeben, die sich innerhalb dieser rechtlichen Strukturen sozialarbeitsrechtlich bewegen müssen.

Beim Erwachsenenschutz zeichnen sich ebenfalls solche Tendenzen ab. Zunehmend mehr hat der Gesetzgeber erkannt, dass es nicht nur um Kindeswohlgefährdung und Jugendschutz geht, sondern dass auch Erwachsene zu dem Kreis schutzwürdiger Personen gehören können. Diese Erkenntnis hängt nicht zuletzt mit den offensichtlichen Veränderungen der letzten Jahrzehnte zusammen, die mit dem Übergang zur sog. zweiten Moderne, oft auch Postmoderne genannt, einhergingen und aus sozialwissenschaftlicher Sicht als charakteristisch für unsere heutige Gesellschaft gelten.

## Die Merkmale der zweiten Moderne

Nach Ulrich Beck, aber auch einer Vielzahl anderer Gesellschaftsanalytiker*innen, zeichnet sich die heutige Zeit in erster Linie durch Individualisierung aus. Individualisierung beschreibt ein makrosoziologisches Phänomen und bezieht sich auf bestimmte subjektiv-biographische Aspekte des Zivilisationsprozesses, insbesondere der Modernisierung (Beck 1986, S. 206). Es geht darum, dass sich das moderne Leben sichtbar verändert hat, und das in nahezu allen Bereichen. Solche Veränderungen lassen sich natürlich auch empirisch messen. Im Folgenden werden die Veränderungen in einigen ausgewählten Wirkbereichen (vgl. Rau et al. 2013, S. 160 f.) und ihre Bedeutung für die sozialarbeiterische Praxis näher beschrieben, bevor der konkrete Bezug zum Erwachsenenschutzrecht erörtert wird. Ausgewählt wurden vor allem jene Wirkbereiche, die eine weitverbreitete Entwicklungstendenz abbilden (statistische Relevanz) und zudem auch für die Zusammenarbeit relevante Informationen ermöglichen. Vergleichbar dem Erkenntnisinteresse der Adressatenforschung stellt sich die Frage:

Wer lebt wie (partnerschaftliche und familiäre Formen des Zusammenlebens und Gesundheit), wo, (Aufenthaltsbereich), wovon (Arbeitsmarkt) und wofür (Freizeitbereich und Werte)?

### Partnerschaftliche und familiäre Formen des Zusammenlebens

Bestimmte Prozesse gesellschaftlicher Transformation erschließen sich auch dem*der Nichtwissenschaftler*in schneller als andere. So verweisen zahlreiche mediale Darstellungen in diversen Kontexten auf Veränderungen bei den Formen des menschlichen Zusammenlebens. Kinderlosigkeit wird im Kontext von Rentensicherheit thematisiert, die Einführung der gleichgeschlechtlichen Ehe (2017) verweist auf die gestiegene Akzeptanz homosexueller Beziehungen, überfüllte Altenheime und ein Mangel an benötigten Pflegekräften verweisen auf die demografische Alterung der Gesellschaft (vgl. Kricheldorff 2018, S. 113). Wirft man einen Blick auf die Statistiken, so lassen sich als wesentliche Veränderungen in den Bereichen Ehe und Familie die steigenden Scheidungszahlen, der Rückgang von Eheschließungen und der Geburtenrückgang seit Mitte der sechziger Jahre nennen (Peukert 2019, S. 2). Trotz dieser Veränderungen dominiert bei den Familienformen mit Kindern immer noch das klassische Bild der Kernfamilie, bestehend aus Mutter, Vater und Kind(ern) (Rau et al. 2013, S. 160 f.). Dieses ist auch nach wie vor noch Ziel und Orientierung der Lebensplanung junger Erwachsener (Lück et al. 2017). Während die Kernfamilie aber früher eine ideelle Einheit zwischen Ehe, Hausgemeinschaft, Elternschaft und Verwandtschaft darstellte (Lüscher und Pajung-Bilger 1998, S. 8), zeigt sich heute ein zunehmend weites Spektrum alternativer Familienkonstellationen. Neben nichtehelichen Lebensgemeinschaften und Patchworkfamilien existieren auch Zweitfamilien, Regenbogen-Familien und eine große Zahl Alleinerziehender (vgl. Peukert 2019, S. 10 f.). Durch den Anstieg der Kinderlosigkeit hat sich der Nicht-Familiensektor deutlich vergrößert, so dass es in Bezug auf Reproduktion auch zu einer Polarisierung der Lebensformen hinsichtlich der Kinder (mit Kindern vs. kinderlos) kam (Peukert 2019, S. 228). Darüber hinaus führt auch die Zunahme nichtfamilialer Haushalte mit kinderlosen Ehepaaren, Einpersonenhaushalten, nichtehelichen oder gleichgeschlechtlich-nichtehelichen Lebens- und Wohngemeinschaften zu einer Pluralisierung der Lebensformen^2^.

Eine professionelle familien- und lebensformbezogene Soziale Arbeit hat heutzutage mit völlig unterschiedlichen Settings zu tun und beschäftigt sich u. a. inzwischen zunehmend auch mit Erwachsenen, die ihren Lebensmittelpunkt in einer außerfamilialen Lebensform haben (Kroll et al. 2015, S. 1). Dementsprechend muss der zugrunde gelegte Familienbegriff neu konzipiert werden. Er umfasst nun nicht mehr vorwiegend die (klassische) Eltern-Kind-Konstellation. Die generationale Ordnung wurde um Fürsorgekonzepte gegenüber einem schwächeren Dritten erweitert, so dass nun auch die Betreuung alt gewordener Eltern darunterfällt (ebd.). Zudem entwickelten sich entsprechend den veränderten gesellschaftlichen Anforderungen auch neue Arbeitsbereiche wie z. B. die geschlechtsspezifische Väterarbeit (ebd., S. 91). Verbunden mit dem demografischen Wandel sind nicht nur der Auf- und Ausbau erweiterter Hilfesettings, sondern auch eine verstärkte Orientierung an den Prämissen der Sozialen Gerontologie durch die Soziale Arbeit notwendig geworden (Kricheldorff 2018, S. 113 f.).

### Aufenthaltsbereich

Auch im Aufenthaltsbereich lassen sich Veränderungen aufzeigen, die sich in erster Linie durch das Merkmal einer verstärkten räumlichen Mobilität charakterisieren lassen. Sie ist zum „prägenden Moment moderner Arbeitsmärkte und Gesellschaften geworden“ (Götz et al. 2010, S. 10), und zunehmende Mobilitätsanforderungen erzwingen auch neue Orientierungsformen (Rau et al. 2013, S. 165). Bei der Arbeitsmigration z. B. erschweren v. a. die unterschiedlichen gesetzlichen Rahmenbedingungen und Verwaltungsabläufe innerhalb der EU die soziale Einbindung des*der Einzelnen. Schlechtere Chancen auf eine Teilhabe am Erwerbsleben gehen oftmals einher mit geringeren Partizipationsmöglichkeiten am Gesundheits- und Bildungssystem. Die Ursache hierfür ist ein Konglomerat verschiedener Faktoren, die aus Sicht der Sozialen Arbeit einen mehrdimensionalen Blick erfordern (Schirilla 2020, S. 138). Beratungsstellen müssen sich dementsprechend um unterschiedliche Aspekte wie Existenzsicherung, Wohnen, Sprachkurse oder die Anerkennung von ausländischen Berufsqualifikationen bemühen. Dies verlangt deutlich vielfältigere Kooperationsformen der sozialen Hilfesysteme – mit entsprechender Öffentlichkeitsarbeit, Unterstützung und Austausch. Oder anders gesagt: Zunehmende Mobilität der Menschen erfordert auch zunehmende Mobilität und Flexibilität in der Sozialen Arbeit.

### Arbeitsmarkt

Veränderungen sind auch im Leistungsbereich der Individuen erkennbar. Dies verdeutlicht ein Blick auf die Entwicklung des Arbeitsmarktes. Bis zum Jahr 2019 gab es einen fast 20 Jahre anhaltenden Anstieg der Erwerbstätigkeit in Deutschland. Mit rund 45,3 Mio. lag die Zahl der Erwerbstätigen 2019 auf dem höchsten Stand seit der Wiedervereinigung, bis es pandemiebedingt zu einem Rückgang kam. Neu ist dabei, dass sich durch Corona nicht nur wie bei vorherigen Rezessionen die konjunkturelle Arbeitslosigkeit Geringqualifizierter zu verfestigen scheint, sondern dass auch höhere Qualifikationsgruppen betroffen sind (vgl. Hutter und Weber 2020, S. 42 f.).

Jedoch zeigt ein Blick auf die Art der Beschäftigungsverhältnisse, dass der bloße Anstieg vor der Pandemie nicht als Indikator für eine rein positive Entwicklung gesehen werden kann. Denn der statistische Anstieg war und ist in erster Linie – mit über 20 % – Teilzeitbeschäftigungsverhältnissen geschuldet (Sorger 2020, S. 176 f.). Weitere ca. 12 % sind geringfügig entlohnte Beschäftigte. Daneben gibt es knapp 10 % an Personen, die neben einer sozialversicherungspflichtigen Beschäftigung einem zusätzlichen Nebenjob nachgehen, um ihre Lebenshaltungskosten decken zu können. Bedenkt man, dass es zeitgleich noch zu Beschäftigungsformen wie Leiharbeit, Scheinselbstständigkeit und zeitlich befristeten Arbeitsverhältnissen gekommen ist, so wird deutlich, dass es – ungeachtet der Gesamtbeschäftigung – zu einem Prozess der Umschichtung gekommen ist: weg von dem klassischen Normalarbeitsverhältnis (mit unbefristeter Festanstellung) hin zu sogenannten atypischen Beschäftigungsformen, die in der Regel mit reduzierten arbeitsrechtlichen Standards einhergehen und bei denen die an Normalarbeitsverhältnisse gebundenen sozialen Sicherungssysteme oft nicht mehr hinreichend greifen, was weitreichende Folgen haben kann (Rau et al. 2013, S. 29). Durch die aktuelle pandemische Lage hat sich die Situation sogar noch verschärft. Allein im August 2020 lag die geschätzte Zahl der sich in Kurzarbeit befindenden Personen in Deutschland bei rund 4,7 Mio. (Link und Sauer 2020, S. 68). Das Ausmaß der langfristigen Folgen für einzelne Personen, wie z. B. die Altersversorgung der Erwerbsbevölkerung, hängt von der zukünftigen Entwicklung des Arbeitsmarktes und der Wirtschaft ab (vgl. dazu Geyer 2021, S. 3 ff.). Durch die staatlichen Corona-Maßnahmen sind jedoch auch heute schon viele Angehörige bestimmter Berufsgruppen in eine unerwartete finanzielle Schieflage geraten, deren Folgen sich in ganzer Intensität wohl erst später zeigen werden. Ein vorzeitiger Rückgriff auf individuelle Rücklagen fürs Alter oder der Jobverlust im höheren Alter, der zu einer frühzeitigen Beendigung der Erwerbsleben führt, hat dabei auch Folgen auf die Lebensgestaltung und -qualität im Alter. Diese unvorhergesehene Entwicklung wird insbesondere die Schuldnerberatung vor Herausforderungen stellen. Zwar führte die Reform des Verbraucherinsolvenzrechts mit der Verkürzung des Restschuldbefreiungsverfahrens zu neuen Möglichkeiten, jedoch bleibt das Stigma der Überschuldung mangels einer gesetzlichen Änderung der Speicherfrist für Wirtschaftsauskunfteien nach wie vor mit entsprechenden Folgen an den Betroffenen hängen (Zerhusen 2021, S. 31).

### Freizeitbereich

Tiefgreifende Veränderungen lassen sich bis zum Ausbruch der Pandemie auch im Freizeitbereich erkennen. Seit den 90er Jahren haben die Deutschen erstmals mehr Stunden an Freizeit zu Verfügung, als sie für den Lebenserwerb einsetzen müssen (Opaschowski 2008, S. 32 f.). Dabei ist der Freizeitbereich hinsichtlich seiner Vielfalt und Möglichkeiten größer denn je. Für Berufstätige allerdings verschwimmen seitdem auch die Konturen zwischen Arbeitswelt und Freizeit. Während freizeitorientierte Ansprüche an die Arbeitswelt herangetragen werden, zeichnet sich Freizeit inzwischen verstärkt auch durch einen Arbeits- und Leistungscharakter aus. Bedingt durch die veränderte Lage zu Zeiten der Pandemie hat sich diese Situation noch verschärft. Die staatlichen Schutzmaßnahmen zur Eindämmung des Virus haben in vielen Fällen die Trennung von Arbeit und Privat fast vollständig obsolet werden lassen. Die Verlagerung der beruflichen Tätigkeit ins Homeoffice führte, gleichermaßen wie die häufig notwendig gewordene Rundum-Kinderbetreuung, zu einer starken Vermischung beruflicher und erzieherischer Tätigkeiten und auch dazu, dass das private Lebensumfeld weniger als zuvor zur Erholung dient (vgl. Gimpel et al. 2020, S. 5).

Bei den nicht und vor allem – aus Altersgründen – nicht *mehr* Berufstätigen verhält sich die Lage anders. Durch die höhere Lebenserwartung hat sich für viele die *nachberufliche* Lebensphase deutlich verlängert und wird zu einer großen Gestaltungsaufgabe. Effekte der möglichen Einsamkeit im Alter haben sich durch die soziale Isolation während der Pandemie noch verschärft. Dabei trifft die Gefahr der Vereinsamung nicht nur pflegebedürftige alte Menschen, sondern auch die, die noch bei guter Gesundheit sind und selbst im Rahmen freiwilliger Tätigkeiten aktiv sein wollen, denn auch sie werden in ihren Aktivitäten ausgebremst (Schroeter und Seifert 2020, S. 8). Gerade zu Zeiten der Krise muss die soziale Altersarbeit zwischen den verschiedenen Alterskulturen mit ihren entsprechenden Anliegen differenzieren, um sich ihre bedürfnis- und risikoorientierte Ausrichtung zu bewahren (ebd.). Und auch nach der Krise wird in Tätigkeitsfeldern der Sozialen Arbeit mit älteren Menschen weiterhin (schon bereits aufgrund des demographischen Wandels) die professionelle Unterstützung, z. B. im Rahmen von Seniorenbegegnungsstätten und -freizeiten, an Bedeutung gewinnen.

### Gesundheit

Neben einer Explosion der Freizeitangebote kam es in den letzten Jahren auch zu einer Expansion der Konsummöglichkeiten (Rau et al. 2013, S. 174 und S. 187). Dabei gibt es kaum etwas, das nicht für Geld zu erwerben ist, und das gilt bis zu einem gewissen Grad auch für die Gesundheit.

Ständige Erfolge und Fortschritte in Medizin und Wissenschaft führten dazu, dass man heute Krankheiten früher erkennen, besser behandeln und zum Teil auch schneller heilen kann. Denkt man an Methoden des „social freezing“, der In-vitro-Fertilisation, der Organtransplantation oder der Palliativmedizin – und die damit verbundenen Möglichkeiten der Schaffung bzw. Verlängerung menschlichen Lebens – so ist offensichtlich, dass diese Fortschritte einen tiefgreifenden Wandel erzeugten, der nicht nur ärztliche Handlungskonzepte veränderte, sondern auch Auswirkungen auf die Rolle und die Situation der (oft immer älteren) Patienten genommen hat.

Während sich die medizinische Versorgung *früher* am Gedanken der *Fürsorge* für den*die Patient*in orientierte, ist sie nun häufig von der freien *Selbstbestimmung* der Betroffenen bestimmt (Huber 2012, S. 11). An die Stelle einer paternalistischen Verfügung der Mediziner*innen über den*die Patient*in tritt heute meist eine partizipative Entscheidungsfindung, ein partnerschaftliches Gegenüber von Mediziner*in und Patient*in, aber auch von Pflegekräften und Angehörigen, wobei nun *alle* Seiten Verantwortung tragen (vgl. Bieber et al. 2018).

### Werte

Und auch im Bereich der *Wertorientierung* kam es, wie bei den familialen Lebensformen, zu einem weltanschaulichen Pluralismus. Neben einer Vielzahl medial vermittelter Lebensstile wird auch ein grundsätzlicher Wertewandel offensichtlich: eine verstärke Zuwendung zu einem Komplex von Werten und Einstellungen, die vorrangig nichtmaterielle Ziele beinhalten und mit der Aufwertung der Lebensqualität und einer kosmopolitischen Orientierung verbunden sind (Rau et al. 2013, S. 181). Die Soziale Arbeit, die sich an den „normativen Standards einer Gesellschaft“ (Scheer 2012, S. 8) orientiert, muss in diesem Kontext die eigene Stellung stets hinterfragen und eine reflexive Distanz zum jeweiligen Zeitgeist einnehmen, um nicht zum „Normalitätsrichter der Disziplinargesellschaft“ im Sinne Foucaults zu werden (ebd., S. 6).

## Individualisierung und Erwachsenenschutzrecht

All diese angeführten Veränderungen sind Ausprägungen der zweiten Moderne. Kurz gefasst könnte man mit den Worten von Ulrich Beck sagen, dass es zu einem Individualisierungsschub kam, „in dessen Verlauf (…) die Menschen in einem historischen Kontinuitätsbruch aus traditionellen Bindungen und Versorgungsbezügen herausgelöst und auf sich selbst und ihr individuelles ‚(Arbeitsmarkt)Schicksal‘ mit allen Risiken, Chancen und Widersprüchen verwiesen werden“ (Beck 1986, S. 41).

Was aber heißt dies nun für das Erwachsenenschutzrecht?

Mit diesen gesellschaftlichen Veränderungen und den gleichzeitigen technischen Errungenschaften entstanden und entstehen für das gesellschaftliche Funktionssystem der Sozialen Arbeit immer wieder vielschichtige Herausforderungen.

Dabei ist die Soziale Arbeit in ihrer Entwicklung natürlich nicht vollkommen frei, denn der Gesetzgeber und die Rechtsprechung machen mit dem Erwachsenenschutz eine bestimmte Zielvorgabe, an der sich ebenfalls ein Trend zur Individualisierung ablesen lässt. Er hängt mit einem veränderten Bild in der Gesellschaft hinsichtlich der Art des Umgangs mit erwachsenen Menschen zusammen. Auch und besonders im Erwachsenenschutz ist, so wie auch im Gesundheitswesen (vgl. Klemperer 2006, S. 61), seit vielen Jahren ein erkennbarer Wandel vom Paternalismus zur Selbstbestimmung im Gange. Während früher mit den Rechtsinstituten der Entmündigung, der Vormundschaft und der Gebrechlichkeitspflege schutzbedürftige Personen bloße Objekte der Rechtsfürsorge waren, so veränderte sich dies mit dem Institut der rechtlichen Betreuung. Ziel ist es heute, jedem Menschen, und das gilt nicht nur für geistig oder seelisch kranke Personen, eine größtmögliche Autonomie zuzugestehen und Eingriffe des Staates nur als Ultima Ratio anzuwenden (vgl. Ganner 2007, S. 62 f.). Damit eng verbunden sind die Bestrebungen, vulnerable Personen zu befähigen und bestärken, selbst am Rechtssystem teilzuhaben und rein „ersetzende Entscheidungen“ durch Betreuer*innen wenn immer möglich durch das Konzept der „unterstützenden Entscheidungsfindung“ abzulösen (Bühler und Stolz 2017, S. 167 f.).

Die Reform im Jahr 1992 und der Wechsel hin zum Betreuungsrecht war und ist so auch als (erstmaliger) Ausdruck eines gesetzgeberischen *Perspektivwechsels* zu verstehen. Die Folgen dieser gestärkten Selbstbestimmung lassen sich auch an den neuesten rechtlichen Entwicklungen auf diesem Gebiet erkennen. Denkt man z. B. im Gesundheitsbereich an Begriffe wie *Vorsorgevollmacht* und *Patientenverfügung,* so können diese – unabhängig davon, wie man sie bewertet – als Folge dieser Individualisierungstendenzen auf rechtlicher Ebene gesehen werden. Das Individuum hat nun die Wahl, aber auch die soziale Verpflichtung, mit den neuen Optionen und seiner Handlungsfreiheit umzugehen. Da mit der gestiegenen Wertigkeit von Selbstbestimmung auch höhere Anforderungen an eine autonome Lebensführung verbunden sind, müssen Sozialarbeiter*innen die dafür nötigen Fähigkeiten ihrer Klientinnen und Klientin zunehmend stützen und fördern.

Dies ist bedeutend, denn auch bei einer wertneutralen Betrachtung der rechtlichen Veränderung zeigt sich eine wesentlich bedeutsamere Entwicklung, für die die angeführten Institute nur symptomatisch stehen. Festzustellen ist eine deutlich zunehmende Subsidiarität der staatlichen Instrumente gegenüber der privatautonomen Vorsorge. Der Gesetzgeber selbst bedient sich somit dieser Individualisierungstendenzen. Ob er dadurch gezielt seine Verpflichtungen hinsichtlich der Schutzfunktion für Schwächere in der Gesellschaft an diese selbst oder ihre nächsten Angehörigen abtritt oder an mehr oder weniger private Dritte auslagert, bleibt eine offene Frage.

## Veränderungen und Entwicklungstendenzen der Sozialen Arbeit

Die Soziale Arbeit bewegt sich also in einem Spannungsfeld zwischen geforderter und erforderlicher Selbstbestimmung ihrer Klient*innen auf der einen und einer in der Praxis auszulegenden gesetzlichen Schutzverantwortung des Staates auf der anderen Seite. Dies erfordert ein ständiges Changieren zwischen einer zunehmend schnelleren richtungsweisenden Rechtsprechung – gezeichnet durch eine große Zahl an auslegungsbedürftigen unbestimmten Rechtsbegriffen – und den praktischen Rahmenbedingungen und Grenzen in der täglichen Sozialarbeit. Dabei ist vor allem das Völkerrecht eine treibende Kraft für die Weiterentwicklung nationalstaatlich geprägter Regelungen. So führte z. B. die Debatte um die Bewertung des UN-Fachausschusses über die Umsetzung der 2009 in Deutschland in Kraft getretenen UN-Behindertenrechtskonvention erneut zu kritischen Fragen nach der Qualität unseres Betreuungsrechts im internationalen Vergleich und der grundlegenden Notwendigkeit weiterer Reformen. Als Resultat dieser Debatte und den Ergebnissen entsprechender Studien kann das jüngst verabschiedete Reformpaket des Vormundschafts- und Betreuungsrechts gesehen werden, dessen Ergebnisse im Betreuungsorganisationsgesetz (BtOG) schriftlich fixiert wurden und das im Januar 2023 in Kraft tritt (vgl. Deinert 2020, S. 169). Insbesondere das darin enthaltene neue einheitliche Registrierungsverfahren (§ 23 BtOG) und die Erbringung eines dann geforderten Sachkundenachweises für alle jene Berufsbetreuer*innen ohne Bestandsschutz (nach § 23 Abs 1 und Nr. 2 BtOG) könnte die Qualität der Berufsbetreuung vereinheitlichen und sichern, oder aber in der Praxis zu unerwarteten Folgen führen (z. B. einer sinkenden Zahl rechtlicher Betreuer*innen durch weniger Nachwuchs aufgrund der erhöhten Anforderungen oder einer zunehmenden Standardisierung durch eine vereinheitlichte, geschulte Vorgehensweisen, die der individuellen Lösungsorientierung ggf. widersprechen könnten). Welche Form von Sachkundenachweisen sich in der Praxis etablieren werden, ist ungewiss; klar ist nur, dass es ein „geregelter Berufszugang mit rechtsstaatlicher Garantie“ sein sollte (Deinert 2020, S. 169).

Aber auch in anderen Bereichen stellten und stellen sich immer wieder eine Vielzahl offener Fragen. Je stärker es dabei um die Beschneidung von Grundrechten geht, desto langwieriger gestaltet sich auch der begleitende Prozess der Rechtsentwicklung. Eine solche Entwicklung lässt sich beispielsweise im Bereich der Einwilligung in ärztliche Zwangsbehandlungen aufzeigen.

So hat zwar der Gesetzgeber im Jahr 2013 mit der Neufassung des § 1906 des Bürgerlichen Gesetzbuches eine lange vorhandene Lücke gefüllt, indem er die Zwangsbehandlung von Personen im Rahmen einer geschlossenen Unterbringung geregelt hat. Für ambulante Patient*innen hatte er dies aber zunächst bewusst unterlassen, um Stigmatisierungen in der Öffentlichkeit durch ständige Zwangsmaßnahmen zu vermeiden. Das BVerfG hatte dazu im Juli 2016 entschieden, dass diese Schutzlücke mit der aus Art. 2 Abs. 2 Satz 1 GG folgenden Schutzpflicht des Staates nicht vereinbar sei (Az.: 1 BvL 8/15). Ein Ende Februar 2017 von der Bundesregierung vorgelegter Gesetzentwurf zur Änderung der materiellen Zulässigkeitsvoraussetzungen von Zwangsmaßnahmen sah vor, die Einwilligung in eine ärztliche Zwangsmaßnahme von der freiheitsentziehenden Unterbringung zu entkoppeln und damit diese auch im Rahmen stationärer Krankenhausaufenthalte zu ermöglichen. Dabei sollte zugleich das Selbstbestimmungsrecht der Betroffenen gestärkt werden, indem auch das Instrument der Behandlungsvereinbarung als besondere Form einer Patientenverfügung in den Gesetzestext aufgenommen wurde. Ambulante Zwangsbehandlungen blieben allerdings auch nach diesem Entwurf weiterhin ausgeschlossen (vgl. Stellungnahme des Bundesrates und Gegenäußerung der Bundesregierung, BTDrucks 18/11617, S. 3, 5 f). Mit der letzten Gesetzesänderung vom 17.07.2017 (BGBI. I. 2426) wurde dann in einem weiteren Schritt eine abschließende Rechtsgrundlage für die betreuungsrechtliche Unterbringung (§ 1906 BGB) und für die betreuungsrechtliche Zwangsbehandlung (§ 1906a BGB) geschaffen. Demnach sind auch ambulante Zwangsmaßahmen nach wie vor nicht zulässig. Dies wurde zuletzt am 07. August 2018 erneut durch einen Beschluss der 3. Kammer des ersten Senates am Bundesverfassungsgericht bestätigt (1 BvR 1575/18).

Selbst diese verkürzte Darstellung der Rechtsentwicklung in diesem Betreuungskontext zeigt die Langwierigkeit einer Rechtsentwicklung, gemessen an der Entstehung einer für die Praxis verbindlichen Leitlinie. Denn bedeutsamer als die konkrete rechtliche Ausgestaltung der betreuungsrechtlichen Frage ist für die konkrete Praxis der tatsächliche Umgang mit solch offen thematisierten Regelungslücken. Das Wissen um potenziell anstehende, rechtliche Veränderungen erfordert auch von Betreuenden zunehmend mehr juristische Grundkenntnisse und die aufmerksame Beobachtung der Rechtsentwicklung.

Hinzu kommen weitere grundlegende Fragen, die die Betreuung zunehmend stärker in die Richtung der sachverständigen Begutachtung verschieben. Denn entscheidend ist in jedem Fall, ob eine betreute Person überhaupt zu einer eigenen Einsicht in ihre Krankheit und Behandlungsbedürftigkeit fähig ist. Doch wie können Betreuer*innen den Willen und die Wünsche der Betreuten feststellen? Nimmt der*die Betreuer*in dabei nur eine ausführende Rolle als „rechtliche*r Assistent*in“ ein? Wann ist eine unterstützende Entscheidungsfindung möglich und wann nicht? Ab welchem Maß wird Betreuung zur heimlichen Entmündigung? Wie verändert sich die Entscheidungsfindung bei der Vertretung durch Angehörige?

In einer alternden Gesellschaft, die vor allem durch eine neue Selbstverantwortung und Selbststeuerung hinsichtlich sozialer Einbindung gekennzeichnet ist (Lang 2016, S. 83), stellt sich die Frage, wer überhaupt noch – und wie lange – auf die Beistandsmöglichkeit durch eine*n Ehegatt*in oder Lebenspartner*in zurückgreifen kann und will. Das heißt, für die selbstautonome Vorsorge bedarf es nicht nur der generellen Möglichkeit des Rückgriffs auf eine*n Ehrenamtliche*n, sondern auch deren bzw. dessen grundsätzlicher Bereitschaft. Welcher medizinische Laie traut sich zu – und auf welcher Grundlage – hier für eine geliebte oder fremde Person eine Entscheidung zu treffen? Und wer ist bei der Beratung von Angehörigen dann überhaupt der*die Klient*in? Eine Problematik, die man aus anderen Bereichen wie z. B. der medizinischen Behandlungen von Schwangeren und ihren Ungeborenen bereits kennt (vgl. Hirschauer et al. 2014).

Aber nicht nur im Gesundheitsbereich, sondern auch in anderen Bereichen der Sozialen Arbeit hat sich die Rolle des*der Klient*in verändert. Das zeigt sich deutlich, denkt man an den Bereich der Haftung oder der Schuldnerberatung. In beiden Arbeitsfeldern ist mit den zunehmend älteren Menschen eine weitere große Zielgruppe hinzugekommen. Neben *bürokratischen* Fragen vom Wechsel einer finanziell nicht mehr tragbaren privaten Krankenversicherung bis hin zu rechtlichen Möglichkeiten wie *Privatinsolvenz* und einem *Pfändungsschutzkonto* stellt sich für Betreuer*innen auch immer häufiger die grundsätzliche Frage nach dem Ausmaß der noch vorhandenen Geschäftsfähigkeit ihrer Klient*innen als Folge alterstypischer Krankheiten wie Demenz oder Alzheimer-Krankheit – und wie man damit umgehen kann und muss.

Diese Beispiele verdeutlichen, dass der Erwachsenenschutz niemals vollkommen sein kann, sondern ständig überprüft und weiterentwickelt werden muss und wird.

Mit dem 3. Opferrechtsreformgesetz vom Dezember 2015 verabschiedete der Bundestag das Gesetz zur Stärkung der Opferrechte im Strafverfahren. Darin enthalten ist im neuen § 406g StPO die psychosoziale Prozessbegleitung – eine besondere Form der nichtrechtlichen Begleitung für besonders schutzbedürftige Verletzte im Strafverfahren vor, während und nach der Hauptverhandlung (vgl. Wenske 2017, S. 457). Darunter fallen auch Erwachsene, sofern sie ihre Interessen nicht selbst ausreichend wahrnehmen können. Auch hier entstand ein neues Tätigkeitsfeld für die Soziale Arbeit. Welche Herausforderungen damit in der sozialarbeiterischen Praxis verbunden sind, wird sich noch zeigen. Rechtlich kritische Aspekte beziehen sich z. B. auf die Fragen: Wo fängt erlaubte Prozessbegleitung an und ab wann ist es schon verbotene rechtliche Beratung? Zu welchem Zeitpunkt beginnt die Beratung und was ist, wenn die Scheu des*der Klient*in bereits einer Strafanzeige im Wege steht?^3^

Vieles lässt sich nur beantworten, wenn man neben Veränderungen der Rechtsprechung auch die Anwendung der Rechtsinstitute in der gerichtlichen Praxis in den Blick nimmt. Nicht zuletzt ergab sich die Notwendigkeit der Gesetzesreform von 1992 daraus, dass die ursprünglich als Regelfall des Erwachsenenschutzes vorgesehene Vormundschaft nach Entmündigung durch die zunehmende Anordnung von Gebrechlichkeitspflegschaften in der Praxis schon lange ersetzt wurde (vgl. Heiderhoff und Rademacher 2018, S. 851).

Und auch heute gibt es eine Vielzahl rechtlich konkurrierender Systeme mit einer strukturell angelegten Mehrgleisigkeit von ehrenamtlichen und berufsmäßigen Betreuer*innen, Betreuungsvereinen, Betreuungsbehörden und einem erweiterten Tätigkeitsfeld der sozialen Arbeit, deren Zusammenspiel Synergieeffekte produziert, aber auch nicht intendierte Nebenfolgen erzeugen kann.

Wechselwirkungen entstehen aber auch durch Einflüsse weiterer Rechtsgebiete. So haben z. B. Veränderungen im Bereich des Konsumentenschutzes und des Sozialrechts Einfluss auf den Kernbereich des Erwachsenenschutzes und umgekehrt. Einfachere Rückabwicklungsmöglichkeiten für Verbraucher*innen oder direkte Sachleistungen können dabei gleichermaßen wie ein niedrigschwelliger Zugang zu informellen Hilfen Veränderungen mit sich bringen und ggf. in vielen Fällen rechtliche Stellvertretung vermeidbar werden lassen. Im Hinblick auf eine systemtheoretische Perspektive, die sozialpolitische Intervention in ihrer rechtlichen, ökologischen, ökonomischen und pädagogischen Dimension betrachtet, lässt sich sagen, dass auch hier eine klare Zuordnung in der Praxis zunehmend schwieriger wird. Zumal eine solche Perspektive ohnehin nicht darüber hinwegtäuschen darf, dass gerade im Bereich der Sozialen Arbeit neben einer Analyse der Sachdimension vor allem auch die Sozialdimension im Vordergrund stehen sollte, da gerade das Erleben der Betroffenen entscheidend ist.

## Fazit

Inwieweit der vom Gesetzgeber eingeräumte Vorrang *anderer Hilfen* vor rechtlicher Betreuung, der Eigenvorsorge vor der staatlichen Rechtsfürsorge bzw. der Unterstützung vor der Vertretung in der Praxis so angenommen und umgesetzt wird und in welchem Verhältnis staatlicher und privater Erwachsenenschutz sich entwickeln, lässt sich aufgrund dieser Wechselwirkungen nur schwer vorhersagen. Sicher ist aber, dass nur eine Gesamtbetrachtung, d. h. ein Blick auf diese Systemzusammenhänge, zeigen kann, wo Defizite bestehen.^4^

Für die nähere Zukunft ist zu erwarten, dass sich durch die aufgezeigten Entwicklungen und mit anhaltender Dauer der Pandemie der Fokus der Sozialen Arbeit weiter verlagert. Galten vor Beginn der Pandemie vulnerable Erwachsene als besonders schützenswert, so liegt der politische Fokus inzwischen vornehmlich auf dem Bereich der Kinder- und Jugendarbeit. Eine geplante Intensivierung und der Ausbau der Hilfesystem werden vorwiegend in diesem Kontext diskutiert.

Innerhalb des Erwachsenschutzes kann zudem davon ausgegangen werden, dass die hohen Anforderungen der gestärkten Selbstbestimmung sich nicht ohne Probleme umsetzen lassen und die Praxis weiterhin auf altbewährte und bereits individuell angepasste Strategien zurückgreifen wird. Dies ist insofern zu begrüßen, als der Erwachsenenschutz eine ständig weiterzuentwickelnde Aufgabe darstellt, die nicht standardisiert werden kann und darf.^5^

Zum einen ist dies bereits aus praktischer Sicht nicht möglich, weil schutzbedürftige Erwachsene gerade keine homogene Gruppe darstellen.^6^ Schon für die schwierige Frage nach Handlungs‑, Erkenntnis- und Steuerungsfähigkeit (und damit ggf. der Notwendigkeit einer rechtlichen Vertretung) sind nicht allein die geistigen Fähigkeiten einer Person – die ja auch zeitlich stark variieren können – ausschlaggebend, sondern auch immer die spezifischen Umstände und deren Schutzbedarf. So besteht z. B. bei vermögensrechtlichen Angelegenheiten allein schon aufgrund finanzieller Interessen Dritter eine höhere Missbrauchsgefahr^7^ als im Bereich der medizinischen Angelegenheiten, die durch ein geringeres Eigeninteresse und stärkere berufsrechtliche Vorgaben von Ärzt*innen ohnehin stärker reglementiert sind (vgl. Brucker 2016a, b).

Zum anderen erfordert das deutsche und internationale Recht, dass der Erwachsenenschutz darauf ausgerichtet sein muss, das Selbstbestimmungsrecht der Betroffenen im Rahmen einer personenzentrierten Betrachtung und Betreuung zu wahren (vgl. Thar 2019, S. 60).^8^ Die rechtlich geforderte Einzelfallorientierung setzt eine problembezogene und funktionale Betrachtung voraus; eine Perspektive, die auch die Soziale Arbeit immer wieder vor neue Herausforderungen stellt. Dabei erfordert die gestiegene rechtliche und gesellschaftliche Wertigkeit von Selbstbestimmung höhere Anforderungen an eine autonome Lebensführung. Im Erwachsenenschutz gilt es daher, die für die entsprechende Umsetzung nötigen Fähigkeiten von Klient*innen zu erkennen, entsprechend zu unterstützen und aktiv zu fördern.

Der Erwachsenenschutz ist sicher auf einem guten Wege, aber noch lange nicht am Ende dieses Weges angelangt. Das im Januar 2023 in Kraft tretende Gesetz zur Reform des Vormundschafts- und Betreuungsrechts (vom 04.05.2021) macht deutlich, dass es bald auf verschiedenen Ebenen zu weiteren Veränderungen kommen wird. Wie sich allerdings die Erscheinungs- und Verwirklichungsformen des Rechts dann im sozialen Leben und damit auch in der Sozialen Arbeit auf Dauer entwickeln, hängt von den Anforderungen in der Gesellschaft der Zukunft ab. Mit einer gewissen Sicherheit vorhersagen lässt sich zum aktuellen Zeitpunkt lediglich, dass sich mit diesen neuen Anforderungen das Recht (aber) stärker denn je auch hin zu den Wirklichkeitswissenschaften wird öffnen müssen. Für die Soziale Arbeit und diejenigen, die sie an den Klient*innen zu leisten haben, heißt dies, mit diesen schnelllebigen und immer komplizierteren Entwicklungen Schritt halten zu können. Dies setzt Kenntnisse der und ein Verständnis für die notwendigen – insbesondere rechtlichen – Grundlagen voraus und macht eine Sensibilität für gesellschaftliche Veränderungen unabdingbar.

## Footnotes

1 Zum Wechsel der Anforderungen an die Profession der Soziale Arbeit im Laufe ihrer (Entstehungs‑)Geschichte siehe die Arbeit von Motzke (Motzke 2014).

2 Dazu kritisch Nave-Herz, die eine durch die wissenschaftliche Verkürzung des Familienbegriffs entstandene Überwertung der Thematik diskutiert (Nave-Herz 1997, S. 36 f.), sowie Peukert (2019, S. 137 f.).

3 Eine solche Evaluation zur psychosozialen Prozessbegleitung wird aktuell vom Kriminologischen Forschungsinstitut Niedersachsen durchgeführt (Laufzeit 01.06.2019–31.05.2022).

4 Um eine möglichst hohe Qualität der rechtlichen Betreuung sicherzustellen, wurden in jüngster Vergangenheit durch das Bundesministerium für Justiz und Verbraucherschutz mehrere Studien in Auftrag gegeben (vgl. BMJV 2018a sowie Matta et al. 2018). Hinsichtlich der vorhandenen Fachkenntnisse von Betreuer*innen kommen sie zu guten Ergebnissen (vgl. Nolting et al. 2018). Bemängelt wird jedoch u. a. eine nicht ausreichend ausgeprägte Selbstreflexion des Faches (ebd., S. 562).

5 Zu den Risiken einer manageriellen Standardisierung und Technologisierung der Sozialen Arbeit durch evidenzbasierte Wirkungsforschung siehe die Darstellung von Otto et al. (2010, S. 7 ff).

6 Diese Erkenntnis findet in der Praxis der Sozialen Arbeit auf vielfältige Weise, u. a. unter dem Schlagwort „diversitysensible Beratung“ auch auf praktischer Ebene Berücksichtigung (Hörmann 2019, S. 9.).

7 Vgl. dazu zuletzt die kriminologischen Ausführungen zu Vermögensdelikten in Betreuungsverhältnissen (Meier et al. 2019).

8 Die Möglichkeiten der personenzentrierten Kontaktgestaltung sind vielfältig (vgl. Kosuch 2018, S. 213 ff.).
